# Cardiac Targeting Peptide, a Novel Cardiac Vector: Studies in Bio-Distribution, Imaging Application, and Mechanism of Transduction

**DOI:** 10.3390/biom8040147

**Published:** 2018-11-14

**Authors:** Maliha Zahid, Kyle S. Feldman, Gabriel Garcia-Borrero, Timothy N. Feinstein, Nicholas Pogodzinski, Xinxiu Xu, Raymond Yurko, Michael Czachowski, Yijen L. Wu, Neale S. Mason, Cecilia W. Lo

**Affiliations:** 1 Department of Developmental Biology, University of Pittsburgh, Pittsburgh, PA 15201, USA; ksf23@pitt.edu (K.S.F.); gag44@pitt.edu (G.G.-B.); tnf8@pitt.edu (T.N.F.); nrp30@pitt.edu (N.P.); xux@pitt.edu (X.X.); yijenwu@pitt.edu (Y.L.W.); cel36@pitt.edu (C.W.L.); 2 Peptide Synthesis Facility, University of Pittsburgh, Pittsburgh, PA 15201, USA; yurko@pitt.edu; 3 Department of Radiology, University of Pittsburgh, Pittsburgh, PA 15201, USA; michael.czachowski@chp.edu (M.C.); masonns@upmc.edu (N.S.M.)

**Keywords:** cardiac targeting peptide, cell penetrating peptides, protein transduction domains, mechanism of transduction, bio-distribution, SPECT imaging

## Abstract

Our previous work identified a 12-amino acid peptide that targets the heart, termed cardiac targeting peptide (CTP). We now quantitatively assess the bio-distribution of CTP, show a clinical application with the imaging of the murine heart, and study its mechanisms of transduction. Bio-distribution studies of cyanine5.5-*N*-Hydroxysuccinimide (Cy5.5) labeled CTP were undertaken in wild-type mice. Cardiac targeting peptide was labeled with Technetium 99m (^99m^Tc) using the chelator hydrazino-nicotinamide (HYNIC), and imaging performed using micro-single photon emission computerized tomography/computerized tomography (SPECT/CT). Human-induced pluripotent stem cell (iPSC)-derived cardiomyocytes (CMCs) were incubated with dual-labeled CTP, and imaged using confocal microscopy. TriCEPs technology was utilized to study the mechanism of transduction. Bio-distribution studies showed peak uptake of CTP at 15 min. ^99m^Tc-HYNIC-CTP showed heart-specific uptake. Robust transduction of beating human iPSC-derived CMCs was seen. TriCEPs experiments revealed five candidate binding partners for CTP, with Kcnh5 being felt to be the most likely candidate as it showed a trend towards being competed out by siRNA knockdown. Transduction efficiency was enhanced by increasing extracellular potassium concentration, and with Quinidine, a Kcnh5 inhibitor, that blocks the channel in an open position. We demonstrate that CTP transduces the normal heart as early as 15 min. ^99m^Tc-HYNIC-CTP targets the normal murine heart with substantially improved targeting compared with ^99m^Tc Sestamibi. Cardiac targeting peptide’s transduction ability is not species limited and has human applicability. Cardiac targeting peptide appears to utilize Kcnh5 to gain cell entry, a phenomenon that is affected by pre-treatment with Quinidine and changes in potassium levels.

## 1. Introduction

The Achilles heel of cardiac therapeutics, as well as diagnostics, has been a lack of cardiac-specific vectors. Plasmids require direct injection into the heart muscle, which requires invasive approaches, has delayed expression, and can invoke a local inflammatory response [1]. Viral-based vectors can be made relatively cardiac-specific by using cardiotropic viruses (e.g., adeno-associated virus serotype 9) and using cardiac-specific promoters, but take from days to a week for expression, have either pre-existing immunity or evoke a rapid immune response on first exposure, generating neutralizing antibodies that preclude repeat therapies [2]. Additionally, these approaches are limited to the delivery of nucleic acids only.

The cell plasma membrane is a semi-permeable barrier that is essential to cell integrity and survival, but at the same time presents a barrier to the delivery of cargo. Hence, the ability of certain proteins to cross cell membrane barriers, described over 25 years ago, was met with great enthusiasm. In 1988, two separate groups demonstrated the ability of the trans-activator of transcription (Tat) protein of the human immunodeficiency virus (HIV) to enter cultured cells and to promote viral gene expression [3,4]. Additionally, it was shown that the Antennapedia homeodomain, a homeobox transcription factor of *Drosophila melanogaster*, could enter nerve cells and regulate neural morphogenesis [5]. Mapping of the domains responsible for this transduction ability led to the identification of the first two cell-penetrating peptides (CPPs), Tat, corresponding to the 11 amino acid basic domain of HIV-1 Tat protein, and Penetratin, corresponding to the 16 amino acid third helix of the Antennapedia domain. These findings were followed by a report by the Dowdy Lab showing that the 11 amino Arginine/Lysine rich Tat fused to β-galactosidase, after an intraperitoneal injection, was internalized into multiple cell types including liver, heart, lung, and kidney. This fusion protein even crossed the blood brain barrier, delivering β-galactosidase in a functional form, thus highlighting the potential of these unique peptides as vectors [6].

The very ability of cationic or hydrophobic CPPs to transduce a wide variety of tissue types in vivo limits their clinical utility because of non-specific targeting of all cell types, leading to higher chances of off-target adverse side effects. One way to identify more cell-specific CPPs is through phage display. Phage display utilizes libraries of various lengths and different bacteriophage strains to identify tissue-specific transduction peptides [7]. This technique involves exposing the target cell type or tissue of interest to a large, randomized library of phage, in which one of the envelope proteins has been modified to display peptides of random sequences. The internalized phage can be isolated, expanded and used in subsequent rounds of screening. The strength of this technique is that a priori knowledge of the target is not necessary.

Our prior work entailed a combinatorial in vitro and in vivo phage display methodology utilizing a commercially available M13 phage display library, in order to identify a cardiac targeting peptide sequence [7,8]. This work resulted in the identification of a 12-amino acid, synthetic, mildly basic, non-naturally occurring peptide (NH_2_-APWHLSSQYSRT-COOH) that was capable of targeting cardiomyocytes (CMCs). Due to its ability to successfully cross the cell membrane barrier of CMCs after a peripheral injection, we termed it cardiac targeting peptide (CTP) [8]. Fluorescently labeled CTP was able to transduce normal mouse heart tissue specifically with little uptake by spleen, brain, or skeletal muscle, and minimal uptake by lung capillaries. Kidney glomerular capillaries showed fluorescence at later time points, implying a renal mechanism of the elimination of the peptide [8].

In the current study, we go on to expand on the bio-distribution studies of CTP using CTP labeled with cyanine5.5-*N*-Hydroxysuccinimide (Cy5.5), in order to minimize background auto-fluorescence issues associated with fluorophores of shorter wavelength. As proof of principle, we show an application of CTP to target Technetium 99m (^99m^Tc) to the heart for imaging purposes. We show that fluorescently labeled CTP is rapidly and successfully internalized by human-induced pluripotent stem cell (iPSC)-derived beating CMCs, indicating that our findings are not species-limited to the mouse heart, but they have potentials for human application. Lastly, we undertake studies into the potential mechanism(s) of transduction, and attempt to identify binding partner(s) for CTP.

## 2. Materials and Methods

### 2.1. Peptide Synthesis

Solid phase peptide synthesis of CTP peptides was carried out using standard fluorenylmethyloxycarbonyl chemistry (FMOC). After completion of the CTP peptide chain assembly, the free N-terminus amino group was manually conjugated with Cy5.5 (Lumiprobe Corporation, Hunt Valley, MD, USA) or in preparation for radiolabeling studies, succinimidyl-*N*-Boc-HYNIC (Synchem UG & Co. KG, Felsberg, Germany) using *N*,*N*-diisopropylethylamine/*N*,*N*-dimethylformamide. Dual-labeled CTP was synthesized with 6-carboxyfluorescein (6-CF) at the N-terminus of the full side chain-protected peptide fragment, followed by activation of the C-terminus with diisopropyl carbodiimide/dimethylamino pyridine, allowing for the attachment of 6-amino-1-hexanol via an ester linkage. Trifluoroacetic acid-mediated removal of the side chain protecting groups from the 6-CF-CTP-CO_2_-hexane-NH2 intermediate was followed by solution phase coupling of tetramethyl rhodamine to complete the 6-CF-CTP-CO_2_-hexane-rhodamine peptide analogue. As a control, a 12 amino acid random peptide (RAN; NH_2_-STLMKFCYVEQN-COOH) was generated using a random peptide calculator from the website https://web.expasy.org/randseq/. The expected mass and purity of the final products were confirmed by analytical C-18 high performance liquid chromatography (HPLC) followed by Matrix Assisted Laser Desorption/Ionization and Time of Flight (MALDI-TOF) analysis.

### 2.2. Bio-Distribution Studies

All animal protocols were approved by the University of Pittsburgh’s Institutional Animal Care and Use Committee (No. 18052818). Six to eight-week-old wild-type CD1 mice were injected intravenously with CTP-Cy5.5 or RAN-Cy5.5 at a dose of 10 mg/kg. The peptides were allowed to circulate for 15, 30, 45, 60, 90, or 360 min, after which mice were euthanized, fixed with 3 mL of 10% formalin, various organs dissected out, fixed and embedded in paraffin, and sectioned. The sections were cross-stained with nuclear stain 4′,6-diamidino-2-phenylindole (DAPI), mounted and fluorescent microscopy performed. Fluorescence in an organ was quantified by drawing regions of interest using ImageJ (National Institute of Health, Bethesda, MD, USA). All time-points and peptides were run in triplicate.

### 2.3. Cardiac Targeting Peptide Imaging Studies

Cardiac targeting peptide was synthesized as detailed above, with HYNIC conjugated to the N-terminus and labeled with ^99m^Tc in a manner that was analogous to that described previously [9]. The desired ^99m^Tc-HYNIC-CTP was purified from the crude reaction mixture using solid-phase extraction methods. The radiochemical purity of the final product was determined using reverse-phase HPLC methods.

Wild-type, 6–8 week old, CD1 mice (~25 g) were injected with 1–5 mCi of ^99m^Tc Sestamibi or ^99m^Tc-HYNIC-CTP, and was imaging performed immediately post-injection and at various time points using a Siemens Inveon dual-modality single photon emission computerized tomography/computerized tomography (SPECT/CT) with 5-MWB-1.0 collimators (Siemens Corporation, Washington, DC, USA). The images were resliced for a coronal view and they were Z-stack summed. Analysis of the distribution of the radioisotope was performed using ImageJ software. Measurements were taken by selecting a region of interest at the 0 h time point, applying the same region at different time points for dynamic time course studies. All counts were corrected for the radioactive decay of ^99m^Tc. For a comparison of dynamic planar images, the highest count in each organ (heart, kidney, and bladder) was set at 100%, and the counts at other time-points plotted as a percentage of that count.

### 2.4. Transduction of Human-Induced Pluripotent Stem Cell Cardiomyocytes

All protocols involving human tissue samples were approved by the University of Pittsburgh Institutional Review Board (No. PRO09090021). Fibroblasts from healthy controls were transfected with four plasmids (Addgene: 27077 (pCXLE-hOCT3/4-shp53-F, 27078 (pCXLE-hSK), 27080 (pCXLE-Hul), 27082 (pCXLE-EGFP)) using electroporation and differentiated into iPSCs using standard protocol. Induced pluripotent stem cell status was confirmed by both immunostaining and qPCR analysis of pluripotency markers *OCT4* and *NANOG.* Induced pluripotent stem cell cells were seeded onto Matrix gel pre-coated plates with mTesr1 medium, and then switched to CDM3 media consisting. Approximately 14 days later, beating cells and calcium transient were observed, and cells stained positive for Troponin-T. Beating CMCs were incubated with the dual-labeled CTP (25 μM) for 30 min at 37 °C/5% CO_2_. After the incubation period, cells were washed, and live confocal imaging was performed immediately.

### 2.5. Mechanisms of Transduction

To identify a possible binding partner for CTP, we utilized LRC-TriCEPs technology [10,11] a service provided by Dualsystems Biotech (Zurich, Switzerland). This methodology relies on TriCEPs™, a trifunctional chemoproteomics reagent, containing (1) a N-hydroxysuccinimide (NHS) ester for attachment to the ligand, (2) a protected hydrazine function for capturing the interacting receptor, and (3) a function for the purification of the ligand-receptor complex. Rat cardiomyoblast cell line H9C2 cells were grown in Dulbecco’s Modified Eagle’s Medium: Nutrient Mixture F-12 (DMEM/F12) (Gibco, Gaithersburg, MD, USA; cat #11039) medium supplemented with 10% fetal bovine serum and maintained at ≤70% confluence. A pretest was performed to identify the binding conditions of the TriCEPS-CTP conjugate on H9C2 cells at varying temperatures, times of incubation, and pH, with successful labeling being analyzed by fluorescence-activated cell sorting (FACs). The LRC-TriCEPS experiment was performed with TriCEP-CTP, TriCEPS-transferrin, and TriCEPS-glycine conjugates, each in triplicate. After the incubation period, cells were lazed and purified using solid-state chromatography, and identified using mass spectrometry.

Raw data was converted to mzML using MSconvert (http://proteowizard.sourceforge.net/download.html) and protein fragments searched with COMET-v27.0 (https://github.com/jdswinbank/Comet.git) against UniprotKB (https://www.uniprot.org/) (v57.15, *Rattus norvegicus*). Probability scoring was done with PeptideProphet (http://peptideprophet.sourceforge.net/) and ProteinProphet (http://proteinprophet.sourceforge.net/) of the Trans-Proteomic Pipeline (v4.6.2). Protein identifications were filtered for a false discovery rate (FDR) of ≤1%. Protein fold-changes and their statistical significance between paired conditions were tested using one fully tryptic peptide per protein. Proteins were considered as candidates if they showed a ≥4-fold-change with an adjusted *p*-value of ≤0.05.

The candidate proteins were tested by siRNA knockdown of each in H9C2 cells using a H9C2-specific transfection reagent (Altogen Biosystems, Las Vegas, NV, USA) with scrambled siRNA (Scramb) being used as a negative control. Briefly, ~50 k low passage (<10) H9C2 cells were plated per well of a 6-well plate. Twenty-four hours later, 1 μL of the transfection reagent, 1 μM of siRNA, and 1 μL of condenser reagent were added to 160 μL of DMEM/F12, incubated at room temperature for 30 min, and added to the cells. Thirty-six hours post transfection, cells were incubated with CTP-Cy5.5 (10 μM) and Live-Dead stain (Invitrogen, Carlsbad, CA, USA; cat #L34966), washed, trypsinized and degree of transduction assessed using FACs. H9C2 cells were recovered post FACs analysis to assess for knockdown efficiency. RNA from cells was extracted using RNeasy Plus Micro Kit (Qiagen, Hilden, Germany; 74134), converted to cDNA using the High-Capacity RNA to cDNA Kit (ThermoFisher, Waltham, MA, USA; 4387406), and real time PCR was performed. All data were normalized to β-actin and expression relative to the Scramb sample. H9C2 cells were also pre-treated with Quinidine (10 μM, 20 μM), an inhibitor of Kcnh5, for 6 h, followed by incubation with CTP-Cy5.5 (10 μM) and FACs analyzed. All experiments were performed in triplicate.

To further study the CTP’s mechanism of transduction, CMCs were isolated from 1–3-day old mouse pups using published protocols [12]. Beating CMCs were incubated with the dual-labeled CTP (25 μM) under normokalemic (5 mM) and hyperkalemic (20 mM) conditions. Subsequently, hyperkalemic CMCs were pre-treated with Verapamil (25 μM) or digoxin (100 nM) prior to their incubation with CTP. The degree of transduction of these cells was quantified using confocal microscopy. Cell death was ruled out by restoring normokalemia and observing the resumption of beating. Rhodamine with an ester link was used as a negative control.

### 2.6. Statistical Analysis

All continuous data were analyzed for normal distribution by performing the kurtosis and skewness test for normality. Normally distributed data was analyzed with either the Student’s *t*-test (for two groups) or using analysis of variance (ANOVA). Non-normally distributed data were analyzed using the Wilcoxon Rank-sum test or the Kruskal–Wallis test. A two-tailed *p*-value of <0.05 was considered to be significant. 

## 3. Results

### 3.1. Bio-Distribution Studies

**Figure 1:**
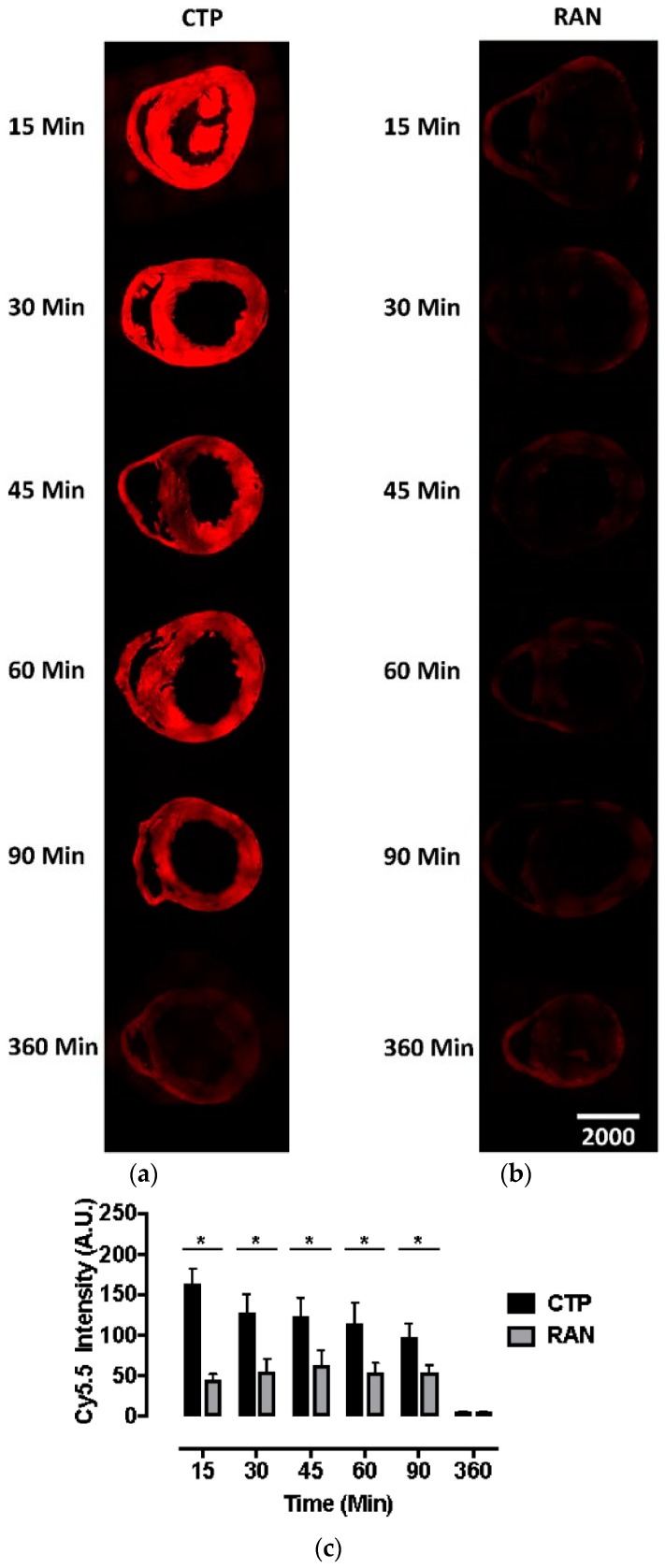
Fluorescence microscopy of murine hearts injected with cardiac targeting peptide-cyanine5.5-N-Hydroxysuccinimide (CTP-Cy5.5; a) or random peptide (RAN-Cy5.5; b) at 10 mg/kg and euthanized at indicated time points. Peak CTP uptake by heart tissue is seen at 15 min with a steady decline over time, both visually (**a**) and by measuring the peak fluorescent intensity (**c**) with almost no fluorescence noted in the RAN-Cy5.5 injected mouse hearts (**b**). All time-points and peptides done in triplicate. Scale bar represents 1000 μm; * represents the two-tailed *p*-value < 0.0001.

Our bio-distribution studies reveal robust and diffuse uptake of CTP-Cy5.5 by the normal mouse myocardium. In contrast, there was very little uptake of RAN-Cy5.5 by the heart (Figure 1). Peak uptake occurred at 15 min, the earliest time-point studied, with steady decline over time. Robust fluorescence was also noted in the liver and kidneys with CTP-Cy5.5-injected mice (Supplemental Figure S1), which decreased over time at this relatively high dose of 10 mg/kg. Neither CTP nor RAN uptake was seen in the brain tissue, indicating that perhaps peptides of this length do not cross the intact blood-brain barrier (Supplemental Figure S2).

### 3.2. Cardiac Targeting Peptide Imaging Studies

**Figure 2:**
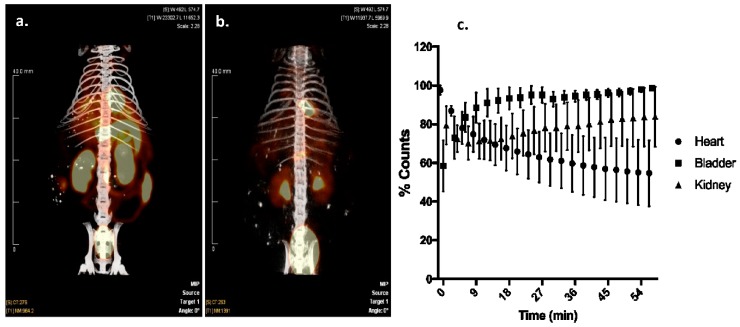
Dual-modality single photon emission computerized tomography/computerized tomography (SPECT/CT) imaging of a mouse injected with ^99m^Tc Sestamibi (**a**), versus ^99m^Tc-HYNIC-CTP (**b**). Sestamibi has a high level of uptake by liver, gut, and kidneys, whereas ^99m^Tc-HYNIC-CTP appeared to be taken up by the heart and excreted by the kidneys. Dynamic planar imaging shows an uptake of ^99m^Tc-HYNIC-CTP by the heart within 5 min of injection, which decreases over time, while bladder and kidney counts go up, implying a renal excretion for the conjugate ((**c**); *n* = 4 in each group).

Cardiac targeting peptide is a cell-penetrating peptide that is able to transduce healthy murine CMCs after a peripheral injection. As a first application of this novel cardiac vector, we explored its ability to deliver ^99m^Tc, a radioisotope to the heart for imaging purposes. Cardiac targeting peptide was synthesized with HYNIC conjugated to its N-terminus, and this construct was labeled with ^99m^Tc (Supplemental Figure S3). An average of 68% (±17%) of the starting radioactivity (56.7 ± 24.3 mCi) could be incorporated into 11.7 μg (±2.8) of HYNIC-CTP (Table 1). This rose to a radiochemical purity of consistently >94% after HPLC purification (*n* = 9; Table 1, Supplemental Figure S4). Wild-type mice were imaged using dual modality microSPECT/CT after injection with ^99m^Tc Sestamibi, the gold-standard formulation of ^99m^Tc used in current clinical practice. As expected, there was cardiac uptake as well as significant uptake by the liver, gut, and kidneys with ^99m^Tc Sestamibi (Figure 2a). In contrast, ^99m^Tc-HYNIC-CTP was taken up by the heart as early as 5 min post-injection (earliest time point at which imaging was possible) with a later increase of counts in the kidneys and bladder (Figure 2b,c). There was almost no uptake seen in the liver or gut.

### 3.3. Transduction of Human iPSC Cardiomyocytes

**Figure 3:**
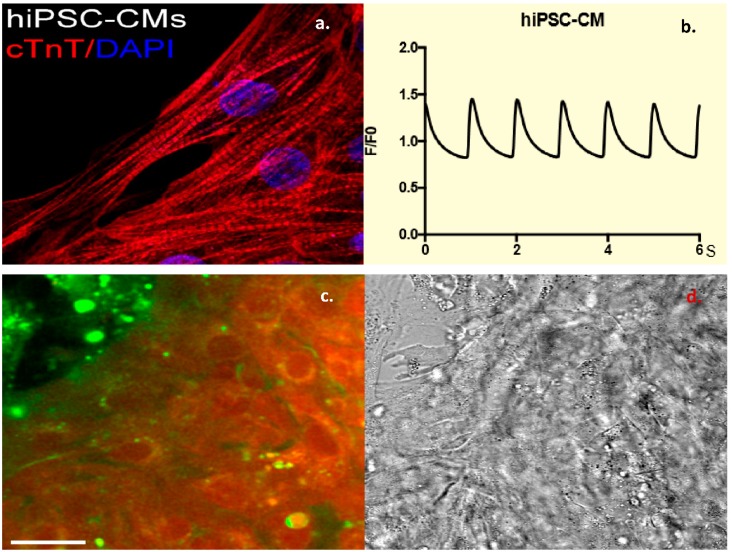
Dual-labeled CTP (25 μM) successfully transduces human-induced pluripotent stem cell (hiPSC)-derived beating cardiomyocytes (CMCs). Human-induced pluripotent stem cell-derived CMCs were confirmed by staining positive for Troponin-T (**a**), and showing cycling calcium transients (**b**). Robust uptake of dual-labeled CTP was seen by these CMCs, with cells labeling red, proving that CTP is internalized and that the rhodamine is cleaved off by intracellular esterases (**c**). Bright-field image showing cell architecture (**d**). Scale bars represent 30 mm. cTnT: cardiac Troponin-T; DAPI: 4′,6-diamidino-2-phenylindole.

Fibroblasts were obtained from nasal scrapes from normal, healthy controls. These were de-differentiated into iPSC cell lines, which were than programmed into beating CMCs. These cells were confirmed to be CMCs by staining for Troponin-T, a cardiac marker (Figure 3a) as well as by the measurement of cardiac calcium currents (Figure 3b). Incubating these cells with dual-labeled CTP showed robust labeling of the beating CMCs with rhodamine. This is proof that CTP was indeed internalized, and the ester link was broken by intracellular esterases, leading to the red labeling of the beating CMCs (Figure 3c), as these esterases are intracellular enzymes and not found in the extracellular space or within the cell membrane. To confirm the internalization of CTP, these cells were also incubated with CTP-Cy5.5 and showed a robust uptake of the peptide at 30 min compared to RAN-Cy5.5 (Supplemental Figure S5). These human iPSC-derived CMC studies show that CTP is not limited in its transduction abilities to the mouse heart alone, but it has the potential for human application, and that CTP is indeed internalized. 

### 3.4. Mechanism of Transduction

**Figure 4:**
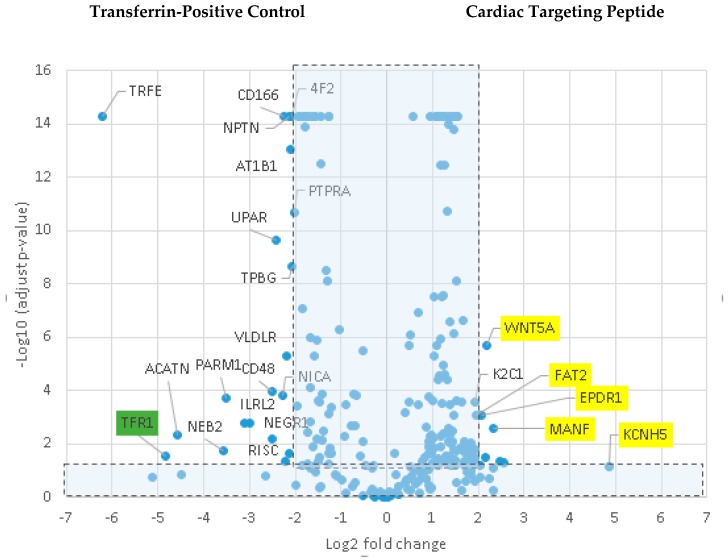
Comparison of transferrin vs CTP-peptide ligands. Volcano plot of proteins identified by mass spectroscopy in the transferrin positive controls versus CTP. The transferrin receptor is identified in the positive control as being almost 5-fold increased over CTP. In CTP samples, Kcnh5, Manf, Epdr1, Fat2, and Wnt5a were increased ≥4 fold with an adjusted *p*-value < 0.05.

**Figure 5:**
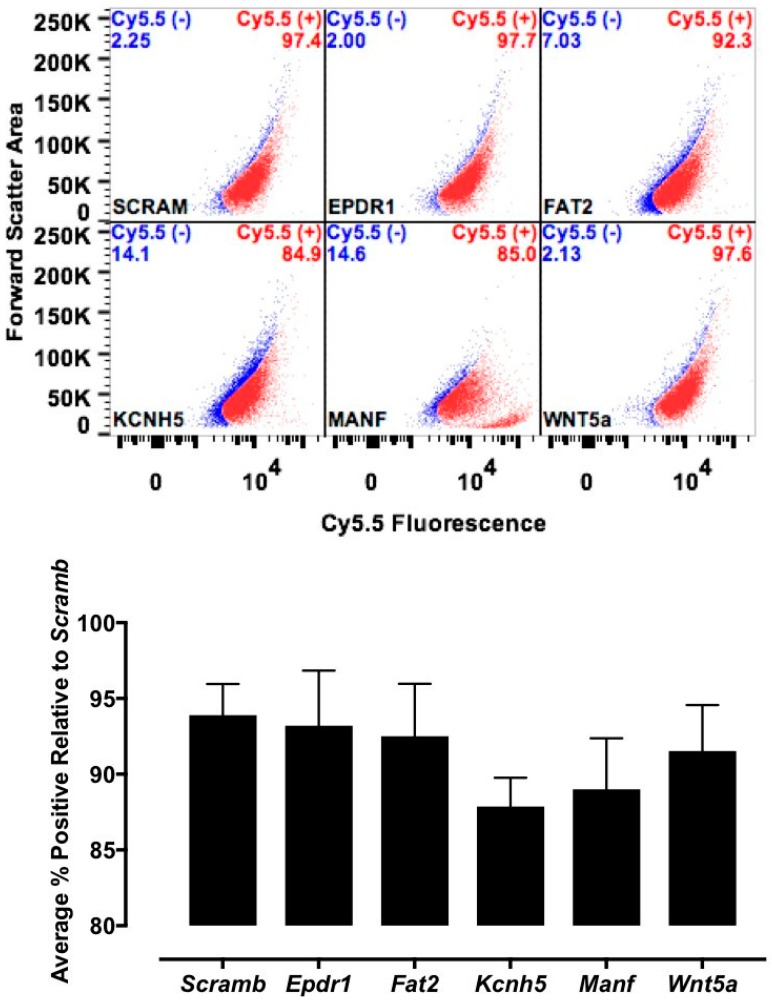
Fluorescence-activated cell sorting (FACs) results after siRNA knockdown. H9C2 cells had the five candidate genes knocked down using rat siRNA and scrambled siRNA (Scram) serving as negative control. Thirty-six hours post-knockdown, cells were incubated with CTP (10 μM) and FACs sorted. A decrease in positively labeled cells of ~10% over Scram is seen only in cells knocked down for *Kcnh5*, showing only a trend (*p*-value = 0.11).

**Figure 6:**
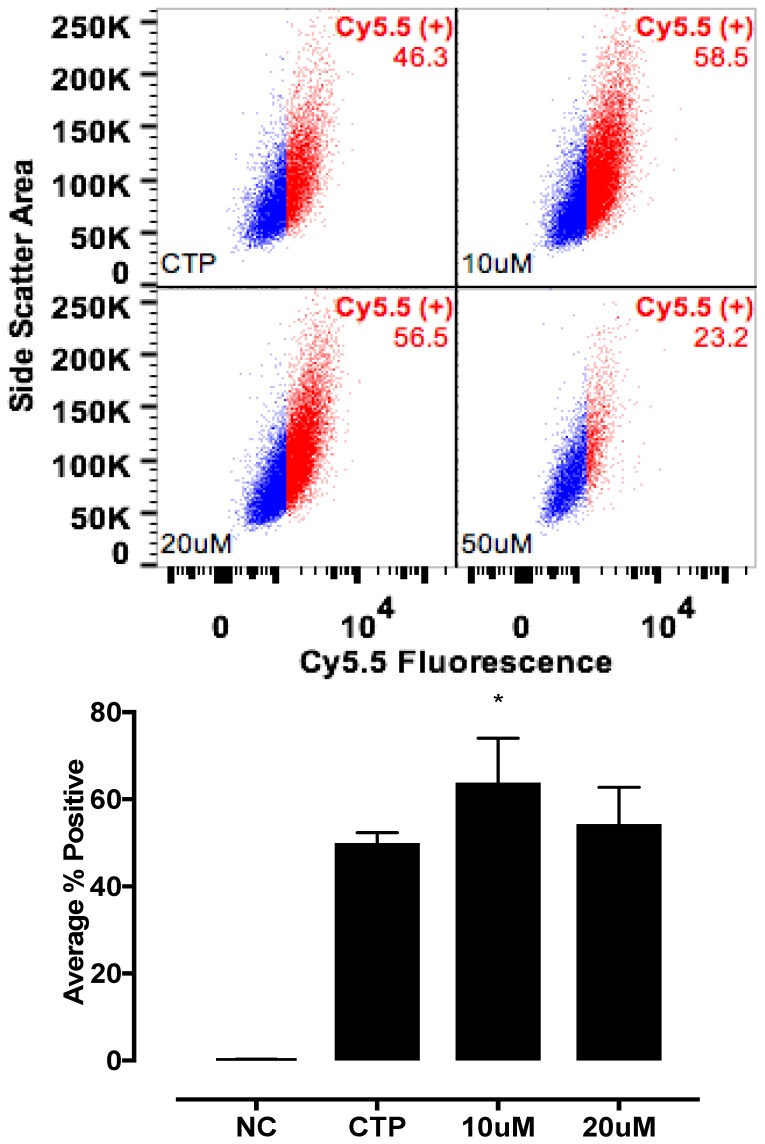
Enhanced transduction with CTP with quinidine, pre-treatment. H9C2 cells were pretreated with various concentrations of Quinidine for 6 h, and incubated with CTP-Cy5.5 (10 μM) for 30 min, washed, trypsinized, and the FACs analysis was performed. A significant increase in transduction was seen with quinidine pre-treatment. NC corresponds to negative control or untreated cells. * Denotes *p* < 0.05 compared to the CTP.

**Figure 7:**
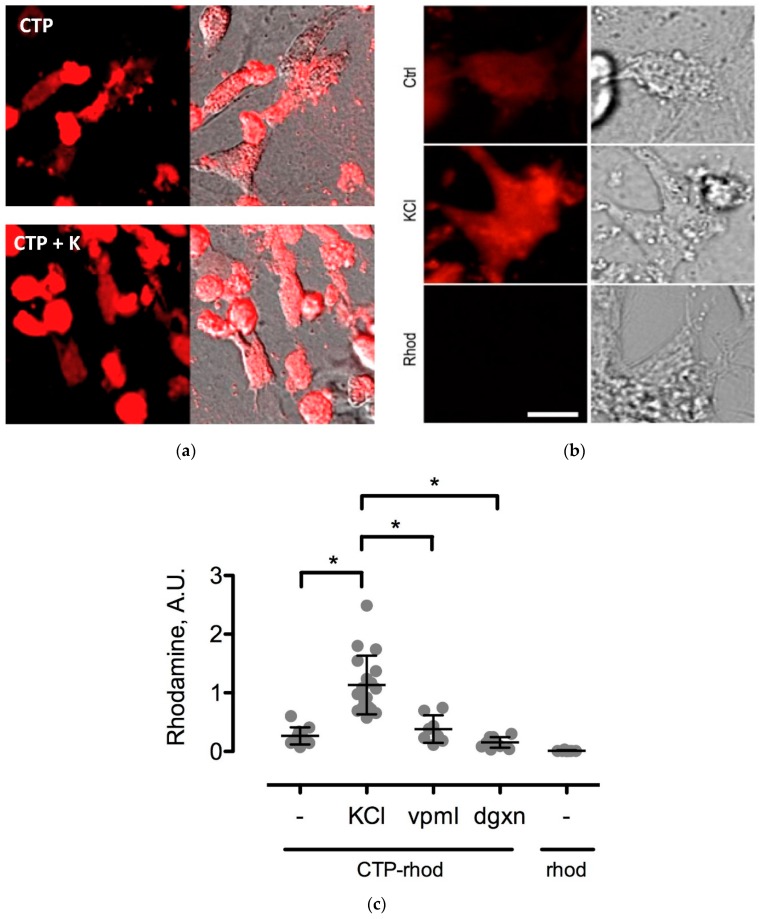
Changes in CTP-rhodamine uptake by changes in extracellular potassium (K^+^) concentrations. Uptake of CTP (25 μM) by CMCs is enhanced by an increase in extracellular K^+^ (**a**; increasing from 5 mM—top panel in **a**, to 20 mM—bottom panel in **a**). Higher magnification views of beating CMCs with normokalemic (Ctrl-5 mM), hyperkalemic (KCl) and Rhodamine (Rhod) as negative control (**b**). This increase was abolished by treating cells with a Na-K^+^ ATPase inhibitor, digoxin (dgxn—100 nM), and a calcium channel blocker, Verapamil (vpml—25 μM) (*n* = 3 for each group). Rhodamine alone (Rhod) was used as a negative control (**c**). * denotes *p*-value < 0.001.

Various cell culture conditions (temperature, pH, and time of incubation) were tested to optimize the labeling of H9C2 cells with a TriCEP-CTP ligand. The optimized cell conditions (Supplemental Figure S5) were used for subsequent testing and mass spectroscopy. Utilizing TriCEPs technology, five candidate proteins were identified that were upregulated at least 4-fold or more, similar to the transferrin receptor, the positive control. These were Epdr1, Fat2, Kcnh5, Manf, and Wnt5a (Figure 4). Figure 4 is a volcano plot comparing mass spectroscopy results with CTP versus transferrin. The positive control, transferrin receptor (Tfre), is highlighted in green with the CTP’s putative binding partners being highlighted in yellow. Of these, Epdr1 is a type II transmembrane protein similar to cell adhesion-binding proteins of the proto-cadherin and ependymin families. Fat2 is a member of the cadherin superfamily, a group of integral membrane proteins, while Kcnh5 is a voltage-gated potassium channel. Wnt5a, a member of the Wnt signaling pathway, is present both on cell membranes and excreted, and it serves as a ligand for Frizzled-5, as well as the orphan tyrosine-kinase receptor. siRNA knockdown of these genes in H9C2 cells, followed by incubation with CTP-Cy5.5 and FACs analysis, showed a downregulation of transduction by ~10% in the *Kcnh5* knockdown cells (Figure 5). No change in the CTP-Cy5.5 labeling of these knocked-down H9C2 cells was seen with *Epdr1*, or *Fat2*. Real-time PCR analysis of these cells showed successful knockdown by ~40–80% compared to the Scramb siRNA (negative control) of all genes except *Fat2*, which we were unable to be successfully knocked down (Supplemental Figure S7). To further test the role of *Kcnh5*, H9C2 cells were pre-treated with Quinidine for 6 h, a Kcnh5 inhibitor that locks the Kcnh5 channel in an open position. Quinidine pre-treatment (10 μM) increased the degree of transduction of H9C2 cells by CTP-Cy5.5 by ~40% compared to untreated cells (Figure 6; *p*-value < 0.05). Further experiments in beating neonatal CMCs showed increase in CTP uptake upon increasing the extracellular potassium concentration from 5 to 20 μM (Figure 7). This increase was significantly abrogated by the pre-incubation of cells with Verapamil, a calcium channel inhibitor, as well as Digoxin, a Na-K ATPase inhibitor (Figure 7). 

## 4. Discussion

In our current body of work, we present the quantitative results of more detailed biodistributions of CTP, an application of it as a cardiac-specific imaging agent, and demonstrate that CTP is not species limited in its transduction abilities. We also present studies into CTP’s putative mechanism of transduction. Cardiac targeting peptide belongs to a class of CPPs that were first identified ~30 years ago [3,4] and that have been shown to carry myriad cargoes like other peptides, proteins, siRNA, DNA, nanoparticles, liposomes, and radio-isotopes [13]. Independent investigators have shown the ability to deliver His-tagged full-length Cre-recombinase protein to cells and mouse heart using CTP [14], as well as exosomes labeled with CTP to H9C2 cells in vitro and mouse heart in vivo [15]. The His-tagged Cre-recombinase protein uptake was seen by skeletal muscle in this study [14], which is in contrast to our work as we did not see any skeletal muscle uptake. Avula and colleagues used CTP to deliver photosensitizers to the rat heart in vivo and sheep heart ex vivo for the photodynamic ablation of atrial fibrillation [16]. Their co-culture of human stem cell-derived CMCs as well as fibroblasts showed CMC-specific internalization of the CTP-photosensitizer conjugate, also proving that the ability of CTP to transduce CMCs is not species limited.

Current cardiac vectors in the form of plasmids or viral vectors can only carry genetic material which, among other drawbacks, suffers from a significant delay in expression. This delay in expression makes these vectors far less useful in acute cardiac conditions such as myocardial infarction or ischemia-reperfusion injury, where the window of therapeutic opportunity is limited to ~ 6 h. Our current biodistribution study shows an uptake of CTP peaking at 15 min, which was the earliest time-point studied, demonstrating that CTP has the potential to serve as a viable alternative, by delivering ready-made peptides and proteins of therapeutic potential. This biodistribution pattern is likely going to be slightly different and dependent on the size of the cargo delivered. However, our earlier work utilized CTP that was labeled with biotin at the N-terminus, and conjugated to streptavidin-Alexa 488, a relatively large cargo, still showed peak uptake at 30 min [8]. Although renal labeling was seen in both the Cy5.5 labeled biodistribution studies and planar imaging with ^99m^Tc-HYNIC-CTP, robust liver uptake was only seen at a higher dose of 10 mg/Kg of CTP-Cy5.5, and not with ^99m^Tc-HYNIC-CTP, in which the utilized dose of CTP was ~1–2 μg, as opposed to ~200 μg (for a 20 g mouse) for the bio-distribution studies. This suggests that at higher doses, there is a loss of specificity, and CTP is taken up by hepatocytes and renal tissue, either non-specifically or perhaps as an additional mode of excretion.

Patients presenting with chest pain are frequently risk stratified with stress tests, a common type of which is myocardial perfusion imaging employing SPECT. These studies are one of the commonest forms of imaging, with 2.7 million cardiac SPECT studies performed for Medicare patients in 2015 alone (CMS Chronic Condition Data Warehouse 2015 data). Due to their sheer numbers, these tests are the largest source of patient exposure to medical procedure-associated radiation [17]. Cardiac imaging radio-isotope formulations, like ^99m^Tc Sestamibi, ^99m^Tc Tetrafosmin, and Thallium 201 have borderline cardiac extraction fractions, with the majority of radiation being taken up by organs other than the heart. This results in poor image quality in some patients, and higher radiation exposures than is actually needed to image the heart itself. We therefore hypothesized that by directing the radiation to where it is needed for imaging with CTP, we can potentially reduce the amount of radiation that is necessary for imaging, as well as improving image quality by improving the signal-to-noise ratio. As a first step towards this goal, we successfully labeled CTP at the N-terminus with ^99m^Tc via a chelator, HYNIC. Our imaging studies show cardiac-specific delivery of ^99m^Tc by CTP compared to ^99m^Tc Sestamibi with almost exclusive renal excretion. This would suggest a more rapid mode of elimination of the administered radiation dose, leading to a decreased transit time in the body, and lower radiation exposure levels. Our radiochemistry experiments show that we can deliver adequate amounts of radiation to the heart, similar to current doses of ^99m^Tc Sestamibi being given for clinical imaging, using microgram (Table 1: 7–15 μg) quantities of CTP. To demonstrate that CTP has potentials for human applications, in this current body of work, we expand on our prior work. CTP was able to successfully transduce human explanted heart tissue from patients undergoing heart transplants [18]. In the current work, CTP also transduced iPSC-derived CMCs, proving that indeed it is not species limited, and therefore has a great potential for human applicability. 

The mechanism of transduction of Tat, identified as a CPP ~25 years ago, still remains a matter of considerable debate [19]. Our work utilizing TriCEPs technology as well as in vitro cell culture work, has identified a handful of putative binding partners with siRNA knockdown, and chemical inhibitor Quinidine experiments suggesting Kcnh5 to be the lead candidate. We pretreated H9C2 cells with Quinidine, a Kcnh5-specific inhibitor [20], and saw greater labeling of cells with CTP-Cy5.5. This is explained by Quinidine blocking the channel in an open state. Additionally, our cell culture work demonstrated changes in extracellular potassium levels significantly increased CTP’s uptake. Collectively, this data points to Kcnh5 being a key player in the transduction mechanism. Although Kcnh5 is a potassium ion channel, and therefore hypothesized to be small and unlikely to allow large cargo-like peptides to pass through, the crystallographic structure of this channel remains unknown. Interestingly, a group of investigators using phage display experiments identified CTP as one of five peptides binding to hydroxyapetite bone-like material in vitro [21,22]. These findings suggest that CTP adheres to extracellular apatite-like material as a possible first step towards interaction with membrane proteins. Further confirmatory experiments, perhaps utilizing a different approach like CRISPR CAS9 knockdown of these putative binding partners, are needed to prove or refute our current working hypothesis of CTP utilizing Kcnh5 for cell entry. 

CTP is not the only cardiac-targeting CPP. Investigators have used in vivo phage display to identify peptides targeting skeletal and cardiac muscle in muscular dystrophy mice [23], vascular endothelial cells [24], and ischemic myocardium [25]. Of these, NH_2_-SIGYPLP-COOH [24] has been incorporated into the coat proteins of adenoviral [26], as well as adeno-associated viral vectors [27] with homing to human vascular endothelial cells. The peptide targeting ischemic myocardium, NH_2_-CSTSMLKAC-COOH [25], has no significant homology to CTP (NH_2_-APWHLSSQYSRT-COOH). Additionally, it was specific to ischemic myocardium, but did not transduce normal myocardium [25], limiting its potential to only certain cardiac pathologies. Follow-up studies on this peptide are lacking. 

## 5. Conclusions

In conclusion, we show that CTP is a novel cardiac targeting vector with peak uptake in cardiac tissue appearing as early as 5 min. It appears to be primarily renally excreted. We also show successful labeling of CTP with ^99m^Tc, with specific delivery of the radioisotope to the heart. Further studies are needed to confirm the exact mechanism and involvement of Kcnh5 in cell entry. We believe CTP has the potential to be a novel cardiac vector with myriad applications that will need extensive further studies to define and fully deploy its potential. 

## 6. Patents

Maliha Zahid (University of Pittsburgh, Pittsburgh, PA, USA) along with Paul D. Robbins (Professor, University of Minnesota, Minnesota, MN, USA) hold a patent on the use of cardiac targeting peptide as a cardiac vector (Cardiac-specific protein targeting domain, U.S. Patent Serial No. 9,249,184).

## Figures and Tables

**Table 1:** Crude radiochemical incorporation yields for Technetium 99m (^99^mTc) radiolabeling of CTP using hydrazino-nicotinamide (HYNIC)-tricine methodology.

Reaction Number	CTP Reaction Mass (μg)	Reaction Starting Radioactivity mCi (GBq)	Crude Radiochemical Incorporation	Radiochemical Purity Following Purification *
1	10	25.1 (0.93)	86% *	>98%
2	6.7	25.1 (1.85)	53% *	95%
3	10	50.0 (1.85)	75% *	>98%
4	10	50.0 (1.85)	80% *	>98%
5	13	50.0 (1.85)	78% *	>98%
6	13	70.0 (2.59)	50% ^†^	95%
7	13	80.0 (2.96)	45% ^†^	96%
8	15	100.0 (3.7)	91% ^†^	>98%
9	15	60.0 (2.22)	57% ^†^	96%

* As determined by reverse-phase HPLC; ^†^ As determined by Sep-Pak measurement.

## Supplementary Materials

The following are available online at http://www.mdpi.com/2218-273X/8/4/147/s1. Detailed methods. Supplemental Figure S1: Transduction of heart, lung, liver, and kidney after intravenous injection in mice. Supplemental Figure S2: Transduction of the brain after intravenous injection in mice. Supplemental Figure S3: Representation of the chemical structures of hydrazino-nicotinamide (HYNIC), HYNIC–Technetium 99m, and CTP–HYNIC used for the in vivo mouse cardiac imaging studies. Supplemental Figure S4: Analytical HPLC traces of representative CTP-HYNIC-Technetium 99m radiolabeling before (a) and after purification (b). Supplemental Figure S5: Transduction of human iPSC-derived CMCs. Supplemental Figure S6: Various cell culture conditions tested to optimize the labeling of H9C2 cells with the CTP–TRICEP ligand. Supplemental Figure S7: Quantitative PCR to assess for the success of siRNA knockdown.
