# Supplementary material for: Cardiac Targeting Peptide, a Novel Cardiac Vector: Studies in Bio-Distribution, Imaging Application, and Mechanism of Transduction
Source: Biomolecules. 2018 Nov 14;8(4):147. doi: 10.3390/biom8040147 (PMC6315548; doi:10.3390/biom8040147)
Supplement: Supplementary file 1 [file biomolecules-08-00147-s001.pdf]

Cardiac Targeting Peptide, a Novel Cardiac Vector: Studies in Bio-Distribution, Imaging Application, and Mechanism of Transduction

Maliha Zahid, Kyle S. Feldman, Gabriel Garcia-Borrero, Timothy N. Feinstein, Nicholas Pogodzinski, Xinxiu Xu, Raymond Yurko, Michael Czachowski, Yijen Wu, Neal S. Mason, Cecilia W. Lo

Supplemental Material

Supplemental Methods

Peptide Synthesis: Solid phase peptide synthesis of CTP peptides was carried out using standard Fluorenylmethyloxycarbonyl chemistry (FMOC) and Oxyma/Ethyl-(2Z)-2-cyano-2-hydroxyiminoacetate/N,N Diisopropylcarbodiimide activation on a Liberty CEM microwave synthesizer. After completion of the CTP peptide chain assembly on Rink Amide-4-*Methylbenzhydrylamine* (MBHA) resin (EMD Millipore), the free N-terminal amino group was manually conjugated with Cyanine5.5-N-Hydroxysuccinimide (Cy5.5-NHS) (Lumiprobe Corporation) or in preparation for radiolabeling studies, Succinimidyl-N-Boc-HYNIC (Synchem UG & Co. KG) using N,N-Diisopropylethylamine/N,N-Dimethylformamide and then cleaved using Trifluoroacetic acid:Water:Triisopropylsilane. The resulting crude CTP peptide conjugates were purified by preparative C-18 RP-HPLC on a Waters PrepLC preparative chromatography system. Expected mass and purity of the final products were confirmed by analytical C-18 RP-HPLC on a Waters Alliance chromatography system followed by MALDI-TOF analysis on an Applied Biosystems Voyager system.

Solid phase peptide synthesis of CTP dual-labeled with 6-carboxyfluorescein (6-CF) at the N-terminus and Tetramethyl Rhodamine at the C-terminus through an ester linkage was carried out on THR-2-Chlorotrityl resin using the Liberty CEM microwave synthesizer and coupling conditions similar to those indicated for the Cy5.5 and HYNIC-CTP analogues. After completion of the peptide chain assembly, the N-terminal amino group was conjugated with 6-CF in DIPEA/TBTU/Hobt overnight at room temperature. Cleavage of the fully side chain protected 6-CF-CTP-OH fragment from the 2-chlorotrityl solid support was accomplished using Hexafluorisopropanol/Dichloromethane (1:3) for 2 hours at room temperature followed by evaporation to dryness on a Buchi Rotavapor system. Activation of the C-terminus of the peptide fragment with Diisopropyl carbodiimide allowed for attachment of FMOC-6-amino-1-hexanol in the presence of Dimethylamino pyridine through an ester linkage. The 6CF-CTP-Ohex-FMOC peptide fragment was then cleaved with TFA:Thioanisole:Anisole:Ethanedithiol (90:5:2:3) for 2 hours at room temperature followed by isolation of the crude product by precipitation in Diethyl Ether and removal of the FMOC group with 20% piperidine in Diisopropyl carbodiimide. The resulting 6CF-CTP-OHex-NH2 intermediate was dissolved in 50% PBS/DMF and conjugated to NHS-Rhodmaine overnight. As control, a 12 amino acid random peptide (RAN; NH_2-STLMKFCYVEQN-COOH) was generated using a random peptide calculator from the website https://web.expasy.org/randseq/. Random peptide was synthesized labeled with Cy5.5-NHS at the N-terminus with amide capping of the C-terminus.

Labeling of CTP with Technetium 99m: CTP was synthesized as detailed above with HYNIC conjugated to the N-terminus. Six to fifteen micrograms of the HYNIC-CTP peptide was incubated with 50mg of tris(hydroxymethyl)methylglycine, 500mL of [Tc99m]TcO4- solution, and 10µl of stannous chloride solution (3.0mg/mL in ethanol) at 95°C for 30mins in a manner analogous to that described previously[9]. The desired Tc99m-HYNIC-CTP was purified from the crude reaction mixture using solid-phase extraction methods. Briefly, the crude reaction mixture was passed across a C8-SepPak Plus (Waters Corporation), the SepPak washed with 10 mL of Milli-Q water and the desired product eluted in 1.5 mL of ethanol. The ethanol was removed under a stream of Argon at 40°C and the product re-solubilized in 0.9% saline for injection. Radiochemical purity of the final product was determined using reverse-phase HPLC methods (Phenomenex Gemini 5 micron NX-C18 column, 4.6 x 100 mm, 110 Å) eluted at a flow rate of 1.0 mL/min under linear gradient conditions.

TriCEPS/LC Mass Spectroscopy: Tryptic peptide fractions were reconstituted in 20µL 5% acetonitrile/0.1% FA/double distilled water, and 1µg per sample was loaded onto an EASY nano-HPLC system (Proxeon) equipped with a RP-HPLC column (75µm x 10.5cm) packed in-house with 10cm stationary phase (Magic C18 AQ 1.9µm, 200Å, Michrom BioResources). The HPLC was coupled to an Orbitrap XL MS (Thermo Scientific) equipped with a nano-electrospray ion source (Thermo Scientific). Peptides were loaded onto the column with 0.1% FA and were eluted with 300nL/min of 99.9% ACN, 0.1% FA. The MS was operated in data-dependent manner, with an automatic switch between MS to MS/MS scans. The five most intense precursor ions were fragmented to acquire MS/MS. Unassigned and singly charged ions were excluded from HCD, and dynamic exclusion was set to 30s.

Raw data was converted to mzML using MSconvert. Fragment ion spectra were searched with COMET (v27.0) against UniprotKB (v57.15, Rattus Norvegicus) containing common contaminants. The precursor mass tolerance was set to 25ppm. Carbamido-methylation was set as a fixed modification for cysteine and oxidation of methionine as a variable modification. Probability scoring was done with PeptideProphet and ProteinProphet of the Trans-Proteomic Pipeline (v4.6.2). Protein identifications were filtered for an FDR of ≤1%. For label-free quantification, proteins were filtered for membrane associated proteins and non-conflicting peptide feature intensities extracted with Progenesis QI (Nonlinear Dynamics). Protein fold changes and their statistical significance between paired conditions were tested using one fully tryptic peptides per protein. Proteins were considered candidates if they showed a ≥4-fold-change with an adjusted p-value of≤0.05.

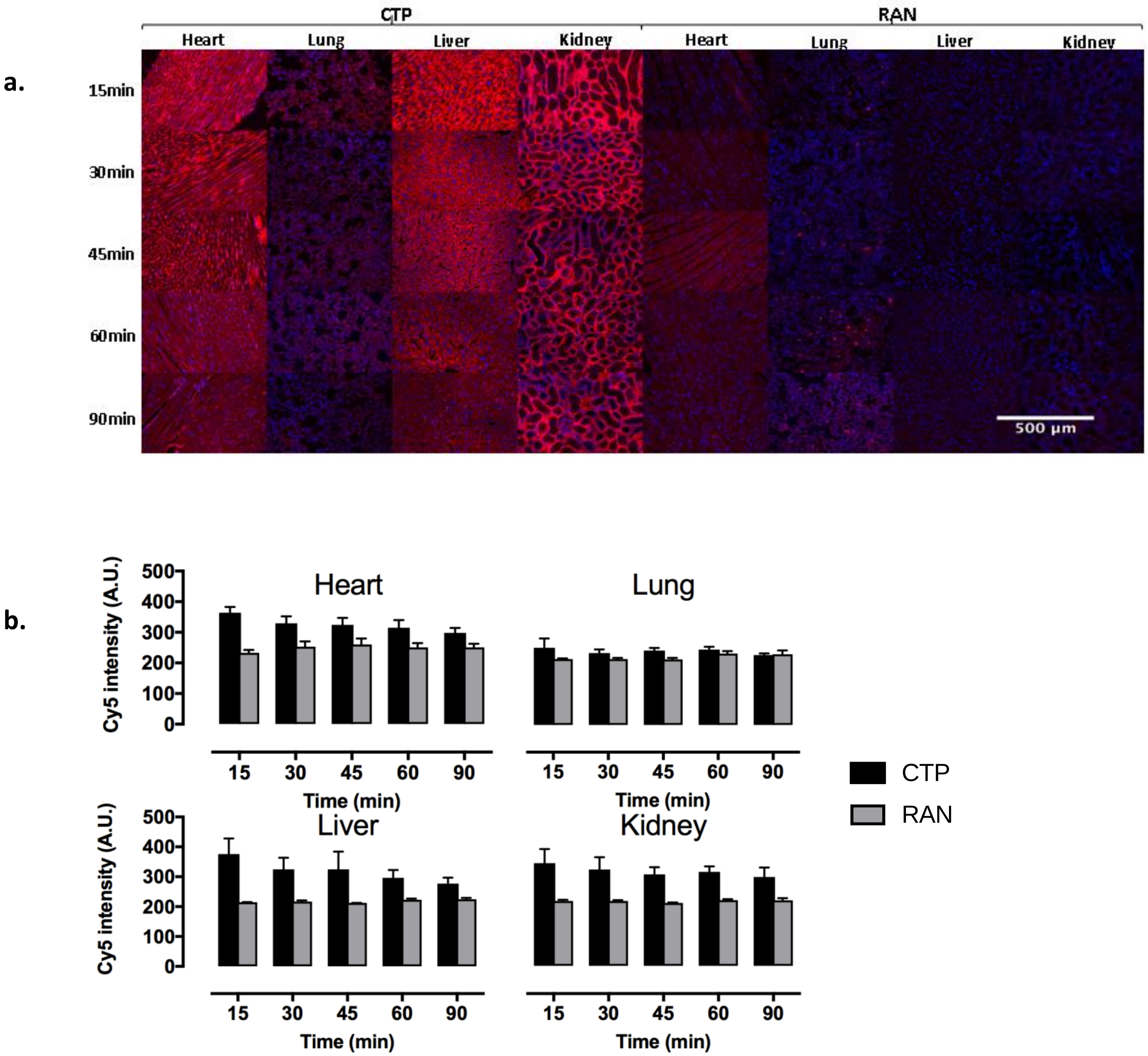

Supplemental Figure 1. Transduction of Heart, Lung, Liver, and Kidney after intravenous injection in mice. Wild-type mice were injected with either CTP-Cy5.5 or RAN-Cy5.5 (10mg/Kg) and euthanized at indicated time points. Peak transduction of heart tissue was seen at 15 minutes with steady decrease in fluorescence over time. Some capillary uptake was noted in the lungs with robust transduction of Liver as well as kidney glomerular capillaries, the latter implying a renal mechanism of excretion (a). Quantification of fluorescent intensity shows significantly increased heart uptake of CTP-Cy5.5 over RAN-Cy5.5. Scale bar represents 500µm.

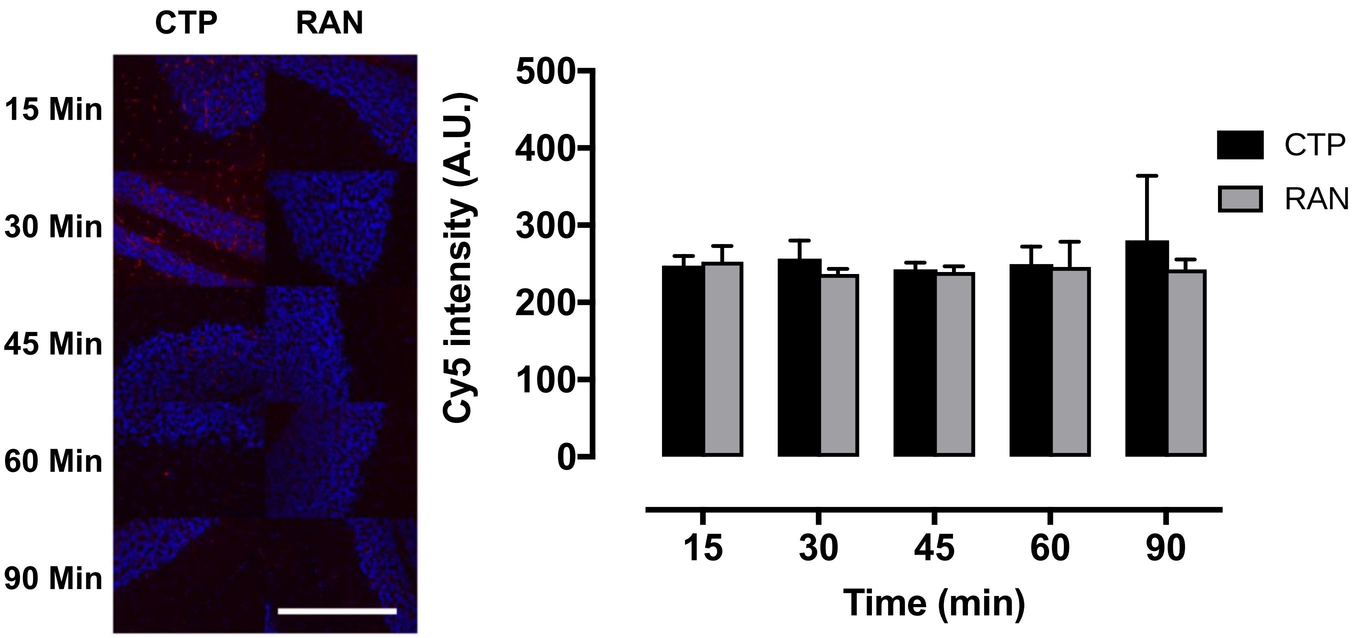

Supplemental Figure 2. Transduction of Brain after intravenous injection in mice. Wild-type mice were injected with either CTP-Cy5.5 or RAN-Cy5.5 (10mg/Kg) and euthanized at indicated time points. No significant uptake of either peptide seen in the brain (cerebellum) region with either CTP or RAN peptide. Scale bar represents 500μm.

HYNIC-Technetium99m

HYNIC

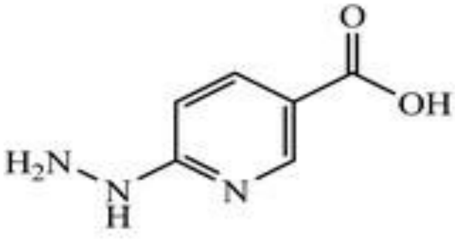

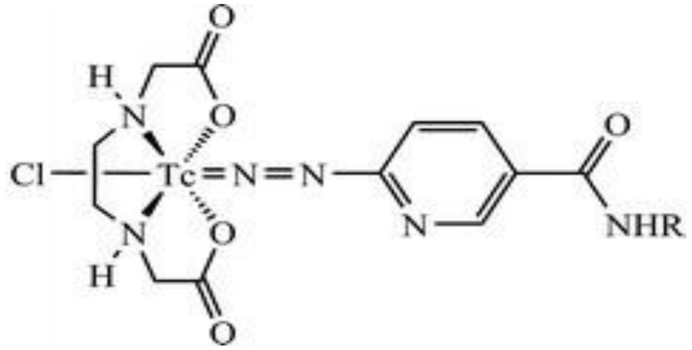

CTP-HYNIC

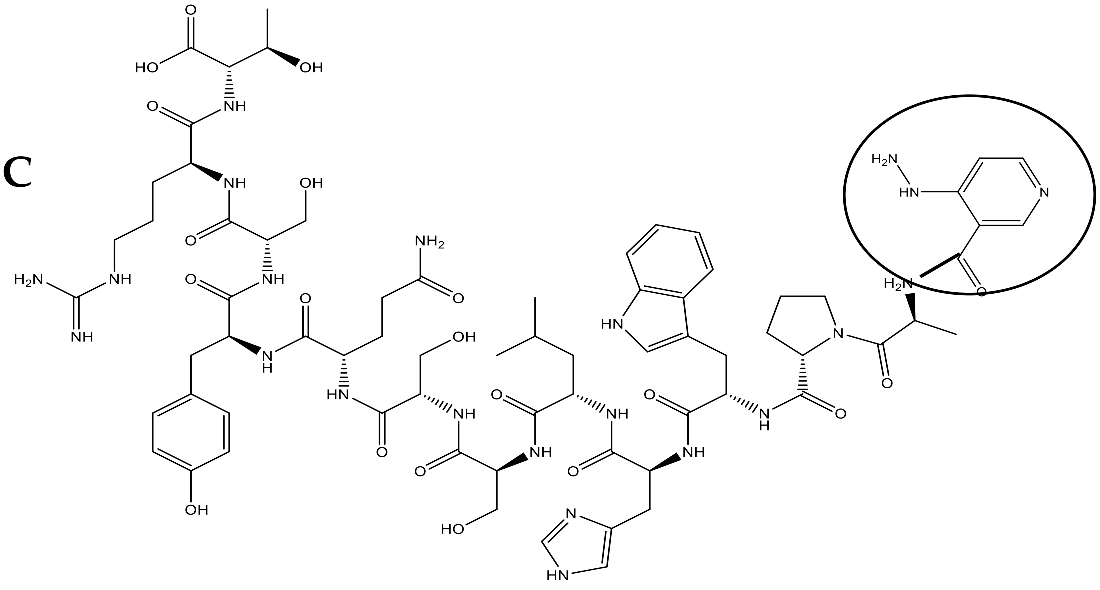

Supplemental Figure 3. Representation of the chemical structures of hydrazino-nicotinamide (HYNIC), HYNIC-Technetium 99m and CTP-HYNIC used for the *in vivo* mouse cardiac imaging studies.

a.

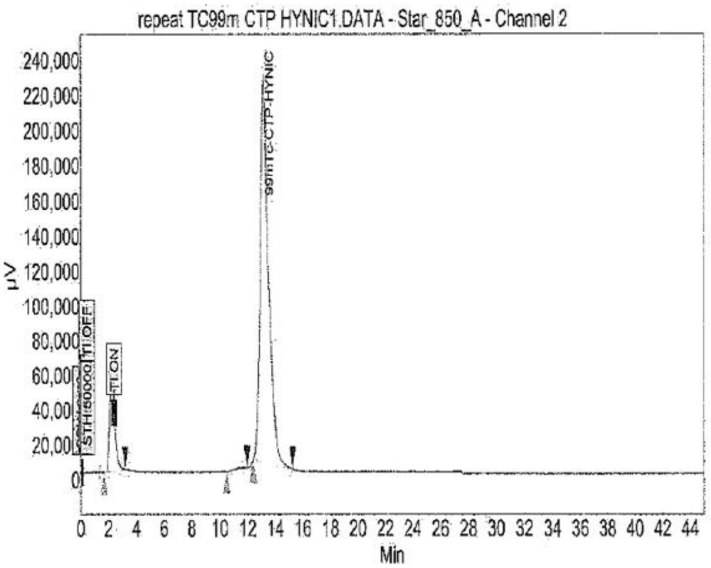

Index	Name	Time [Min]	Area % [%]	Area [µV.Sec]
1	UNKNOWN	2.21	13.036	1374402.4
2	UNKNOWN	11.39	0.316	33303.1
3	99mTc-CTP-HYNIC	13.13	86.649	9135801.8
Total			100.000	10543507.3

b.

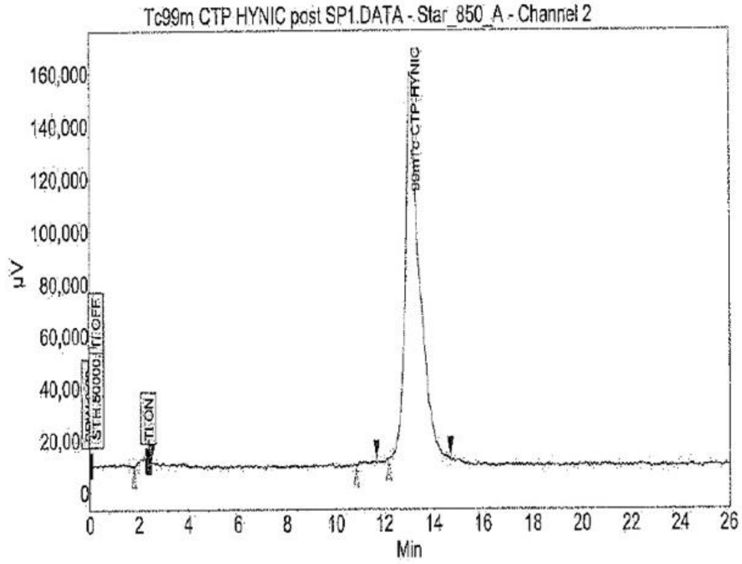

Index	Name	Time [Min]	Area % [%]	Area [µV.Sec]
1	UNKNOWN	2.21	1.037	60233.5
2	UNKNOWN	11.00	0.228	13217.8
3	99mTc-CTP-HYNIC	13.06	98.735	5735168.9
Total			100.000	5808620.2

Supplemental Figure 4. Analytical HPLC traces of representative CTP-HYNIC-Technetium 99m radiolabelings before (a) and after purification (b).

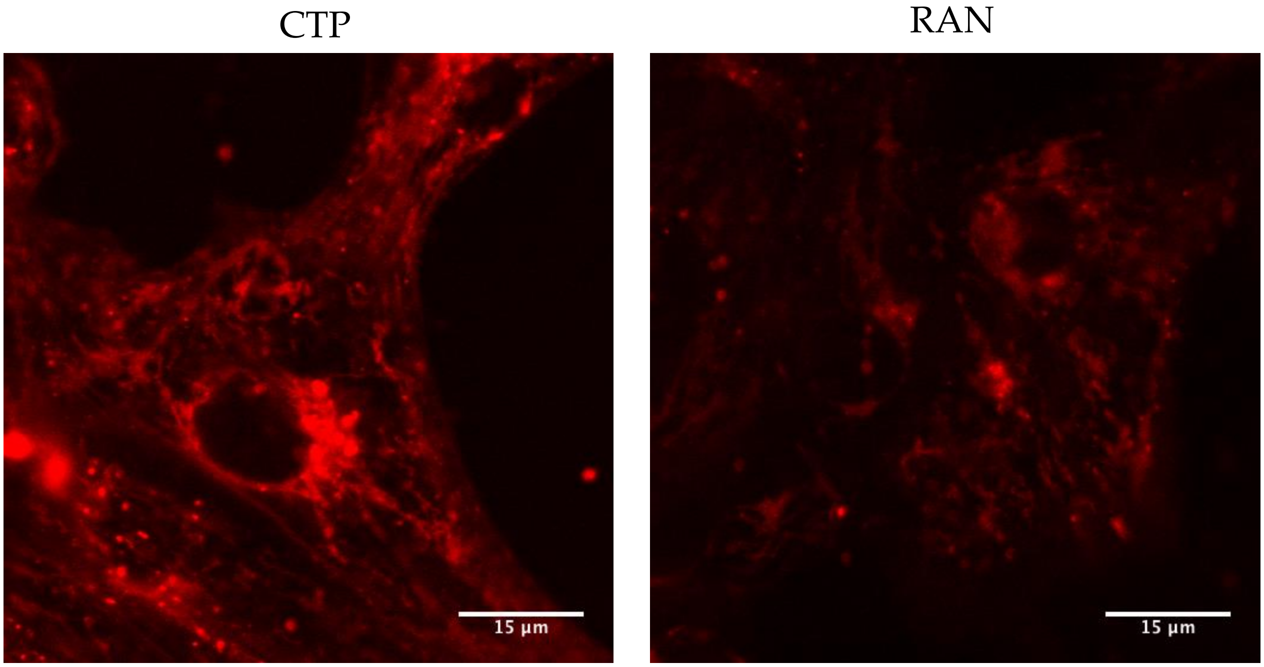

Supplemental Figure 5. Transduction of human iPSC derived CMCs with CTP-Cy5.5 (10µM) or random peptide (RAN; 10µM) for 30 mins at 37°C. Cells were washed 3x with PBS and imaged using confocal microscopy. There is robust uptake of CTP-Cy5.5 by CMCs with some background binding to the gel matrix by RAN peptide.

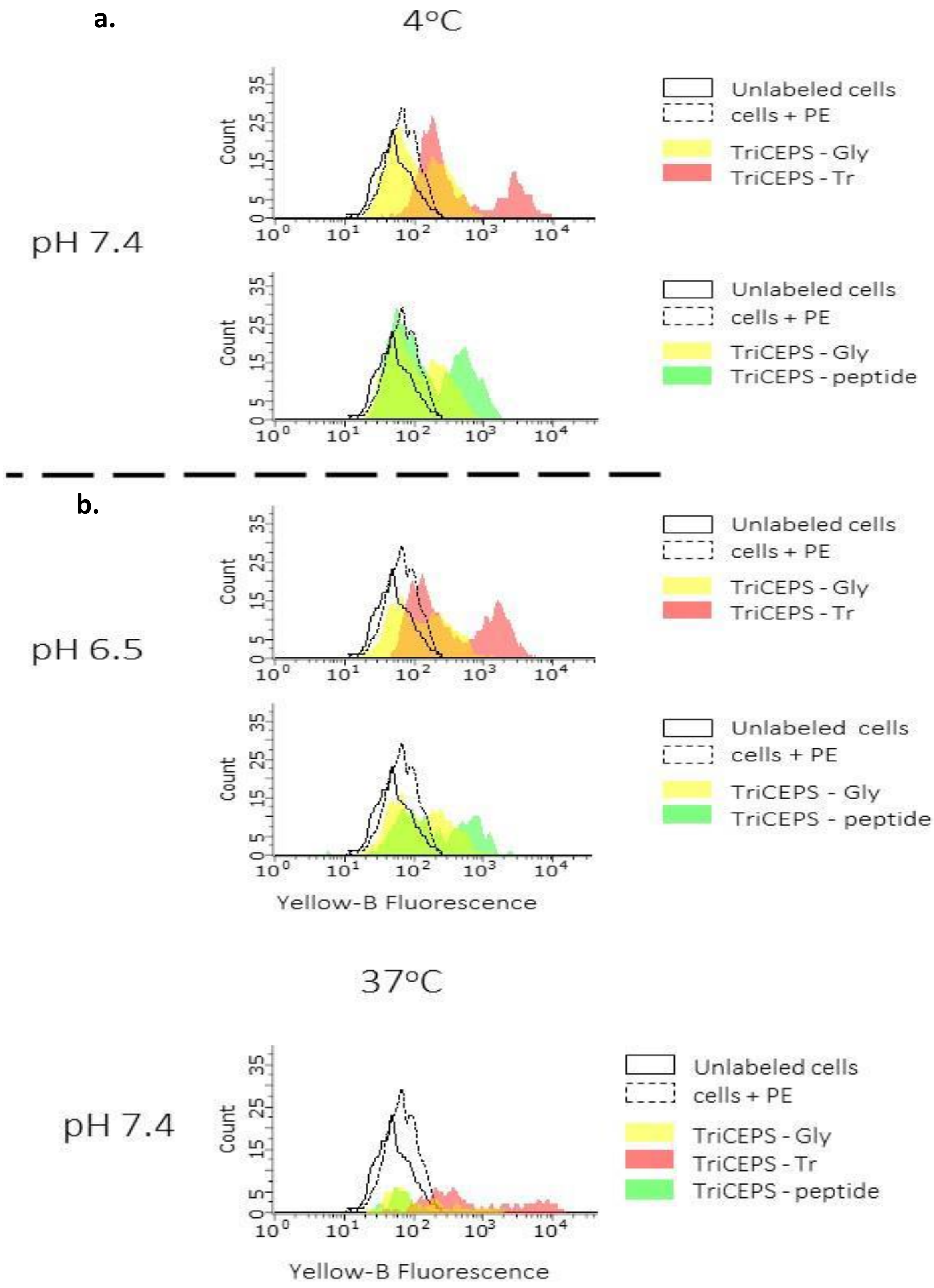

Supplemental Figure 6. Various cell culture conditions tested to optimize labeling of H9C2 cells with CTP-TriCEPS ligand. Optimal labeling of H9C2 cells occurred at 4°C, pH of 6.5 with incubation for 90mins. For the pretest experiments TriCEPS v.2.0 is used. TriCEPS v.2.0 contains a biotin group which reacts with streptavidin conjugated with R-phycoerythrin (streptavidin-R-PE) to determine binding of the ligands to the cell surface by flow cytometry.

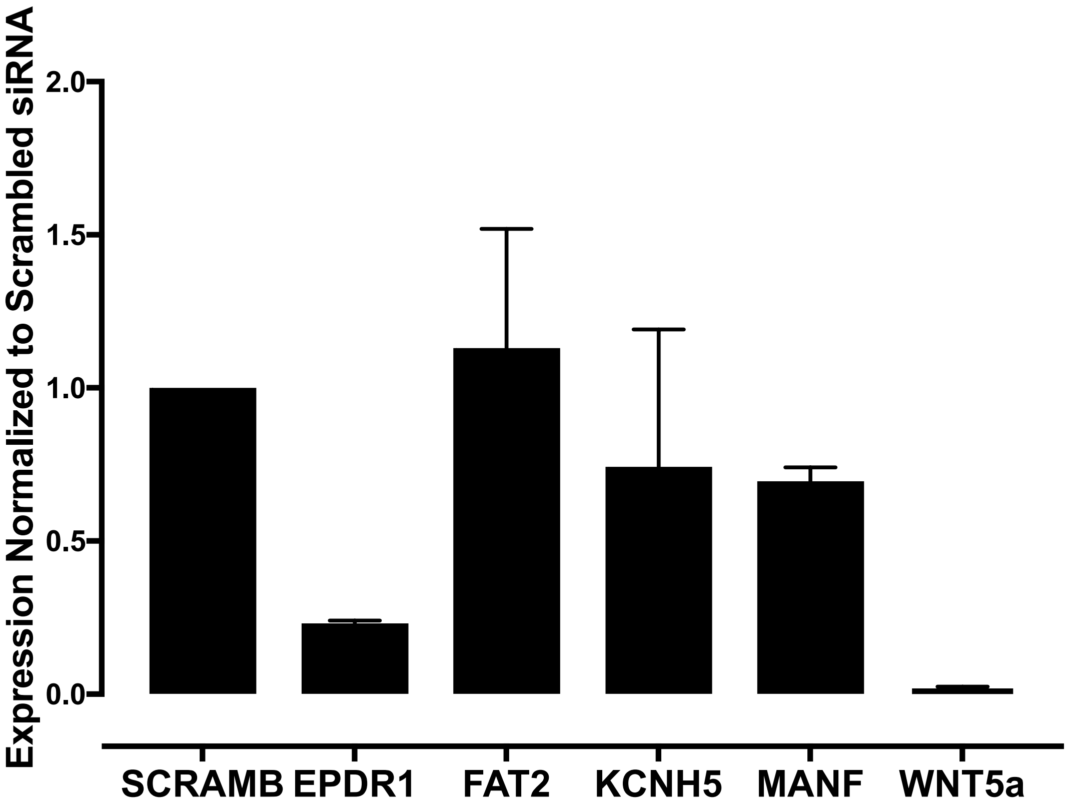

Supplemental Figure 7. Quantitative PCR to assess for success of siRNA knockdown. H9C2 cells were transfected with siRNA for thirty-six hours followed by incubation with CTP-Cy5.5 (2μM) and evaluated by FACs. Cells were collected post-FACs analysis, lysed in RNA lysis buffer, RNA extracted, converted to cDNA and quantitative PCR performed. Data is normalized to scrambled siRNA (Scramb).
